# Vibration Response Imaging: evaluation of rater agreement in healthy subjects and subjects with pneumonia

**DOI:** 10.1186/1471-2342-10-6

**Published:** 2010-03-11

**Authors:** Konstantinos Bartziokas, Christos Daenas, Sebastien Preau, Paris Zygoulis, Apostolos Triantaris, Theodora Kerenidi, Demosthenes Makris, Konstantinos I Gourgoulianis, Zoe Daniil

**Affiliations:** 1 Department of Respiratory Medicine, University Hospital Thessaly, Larissa, Greece; 2 Department of Critical Care Medicine, University Hospital A. Calmette Lille, France; 3 Department of Critical Care Medicine, University Hospital Thessaly, Larissa, Greece

## Abstract

**Background:**

We evaluated pulmonologists variability in the interpretation of Vibration response imaging (VRI) obtained from healthy subjects and patients hospitalized for community acquired pneumonia.

**Methods:**

The present is a prospective study conducted in a tertiary university hospital. Twenty healthy subjects and twenty three pneumonia cases were included in this study. Six pulmonologists blindly analyzed images of normal subjects and pneumonia cases and evaluated different aspects of VRI images related to the quality of data aquisition, synchronization of the progression of breath sound distribution and agreement between the maximal energy frame (MEF) of VRI (which is the maximal geographical area of lung vibrations produced at maximal inspiration) and chest radiography. For qualitative assessment of VRI images, the raters' evaluations were analyzed by degree of consistency and agreement.

**Results:**

The average value for overall identical evaluations of twelve features of the VRI image evaluation, ranged from 87% to 95% per rater (94% to 97% in control cases and from 79% to 93% per rater in pneumonia cases). Inter-rater median (IQR) agreement was 91% (82-96). The level of agreement according to VRI feature evaluated was in most cases over 80%; intra-class correlation (ICC) obtained by using a model of subject/rater for the averaged features was overall 0.86 (0.92 in normal and 0.73 in pneumonia cases).

**Conclusions:**

Our findings suggest good agreement in the interpretation of VRI data between different raters. In this respect, VRI might be helpful as a radiation free diagnostic tool for the management of pneumonia.

## Background

Imaging methods are critical for the diagnosis and management of lung diseases. However, some methods, such as Computed Tomography and radiography, are limited by the fact that they cannot be carried out effectively at the bedside and most importantly that they involve radiation. Other methods, such as ultrasound, use acoustic signals and they do not involve radiation but their diagnostic value meets limitations due to the acoustic damping of the lung parenchyma [1]. In this respect, novel imaging methods based on computer-assisted mapping of lung sounds not complicated by radiation, aim at contributing to the diagnosis of lung diseases.

Vibration response imaging (VRI) is a technique that uses novel technology and measures vibration energy of lung sounds. The principle of the method is based on the capture of the turbulent air and vibrations which are generated within the lungs and airways by the multisensors of the VRI device. Previous reports showed that advancement in lung sound analysis from human-based auscultation to a computer-based analysis tool allows objective and measurable results [2-6].

However, published data regarding the application of the method in patients with lung diseases are sparse. A recent study showed that VRI is a reproducible diagnostic method but included only healthy individuals [7]. Another investigation demonstrated VRI reproducibility in patients mechanically ventilated or in subjects undergoing invasive bronchoscopic procedures [8,9]. Nevertheless, the small number of studies does not allow definitive conclusions for the reproducibility of the technique, especially in the setting of specific disorders which have not been approached.

In the present study, we aimed to evaluate the agreement between different physicians in the interpretation of VRI lung images from healthy subjects and hospitalized patients with community acquired pneumonia.

## Methods

### Study population

The study population consisted of 43 subjects: 23 patients who were diagnosed with community acquired pneumonia and 20 healthy subjects who participated in the study as control cases. Patients were recruited by consecutive sampling from patients hospitalized in the Respiratory Medicine Department of the University of Thessaly between September 2007 and November 2007 and healthy subjects were recruited from the medical personnel of the hospital during the same period.

Diagnosis of pneumonia was determined by the treating physician, based on medical history, physical examination and radiographic findings. Patients with chest cage or spine deformity, skin lesions, excessive hirsutism on the back and any patient deemed unable to be lifted to a near-sitting position with assistance were excluded. Diagnostic and treatment decisions for all cases described in this study were according to accepted criteria for diagnosing pneumonia [10,11]. The study protocol was approved by the Institutional Review Board and informed consent was obtained from each participant prior to the inclusion in the study.

### VRI device

**Figure 1:**
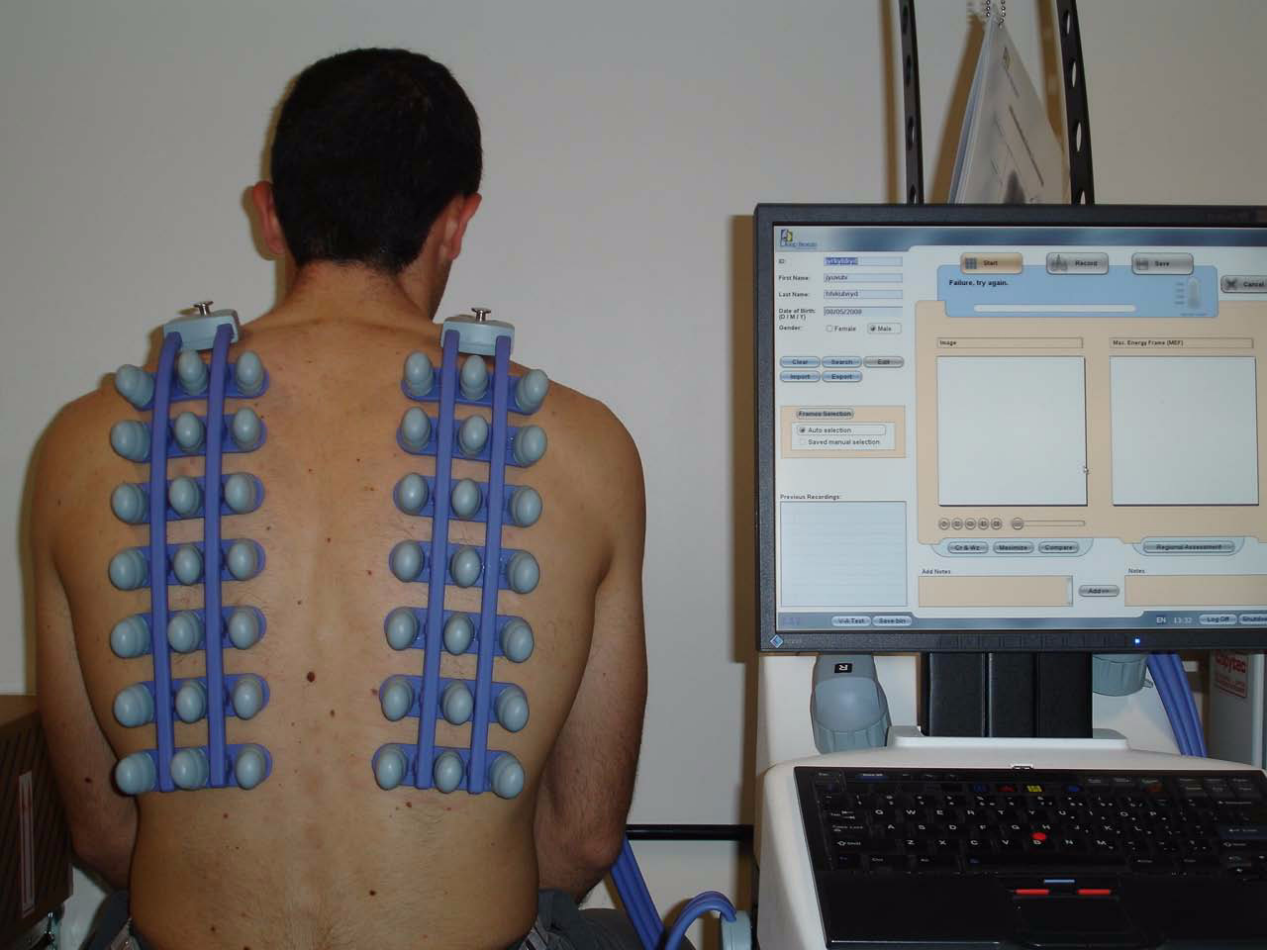
**Attachment of piezoelectric contact sensors of VRI (left and right peripheries of the first row) to the posterior chest**.

Vibration Response Imaging device (Deep Breeze Ltd, Or Akiva, Israel) includes a hardware board for sampling, amplification, processing and A/D data conversion, a PC platform for generation of images and 40 active piezoelectric contact sensors (Meditron, Oslo, Norway) to record lung vibrations. The sensors have a linear frequency response of ± 2 db in the frequency range 50-400 Hz assembled on two planar arrays with a linear frequency response of ± 2 decibel (db) in the frequency range of 50 Hz to 400 Hz and two inactive contact sensors (left and right peripheries of the first row). The arrays were attached to the posterior chest by an open system with a PC-controlled low vacuum to maintain a constant mechanical load on the sensors (Figure 1). The sensors were coupled to the subject's back by a computer controlled low-suction vacuum.

### VRI principles and recordings

All subjects were examined with the VRI at the bedside during their hospitalization and recordings were performed by their attending physician as previously described [12]. Subjects were instructed to breath deeper than normal through an open mouth during a 12-second recording (3 or 4 respiratory cycles). No forced exhalation or other breathing manoeuvres were performed. During air movement in and out of the lungs, vibrations propagate through the lung tissue. Vibrations were recorded by the surface skin sensors, which were attached to the patient's back. The vibration energy was transmitted to the VRI device, and thereafter, a dynamic digital image was created by means of specifically designed software. Lung sound signals were then transferred to a computer and were analyzed by the software. The signals were processed by band-pass filtering with different frequency ranges: 100-250 Hz for capturing breath sounds while filtering heart sounds and chest-wall movement. The VRI dynamic image was created from a series of gray-scale still images similar to ventilation scanning images of the lung or frames which represent 0.17 seconds of vibration energy recording. In addition, a graph is produced that represents the average vibration energy as a function of time throughout the respiratory cycle. High data values, in which lung vibration energy is greater, are depicted as dark colours (black) and low data values are shown as light colours (light grey); the minimum is defined as 'white'. The maximal energy frame (MEF) is the frame producing the maximal geographical area of lung vibrations in the selected range of frames.

### Evaluation of VRI

VRI images were evaluated separately by six raters who were qualified physicians and had undergone a basic training in image interpretation by analyzing healthy learning sample images. The training was conducted by showing to the readers the dynamic images sequentially in order to enhance their ability to distinguish basic characteristics of the VRI image including inspiration, expiration, left right synchronization and peak intensity of inspiration [13].

The raters blindly analyzed VRI findings of the study population in a random order, without any previous knowledge of the participants' medical history or the number of images obtained from them. The raters were blind to one another and evaluated twelve features related to i) VRI graphical quality (very good to excellent or not), ii) synchronization of the progression of breath sound distribution between the left and right lung images, iii-x) localization of abnormally increased or decreased intensity in VRI image in right and left, upper-middle and lower lung zones, xi) presence of artefacts, defined as VRI signal with no or poor progression during inhalation-exhalation and no detection of relevant abnormalities in the Chest X-ray, xii) presence of abnormally high or low intensity on VRI-MEF corresponding to the areas of pulmonary consolidation on chest radiographs. After evaluation of i to x features, patients' and healthy controls' chest radiography was demonstrated to raters and features xi and xii were then evaluated. The participants recorded their interpretation of each VRI on a structured questionnaire and these data were used to evaluate inter-rater agreement. In order to measure intra-observer agreement the evaluation was repeated for all VRI images (thus, each reviewer assessed 86 images in total) using the methodology described above.

### Statistical Methods

For qualitative assessment of VRI images, the rater's evaluations were analyzed by degree of reliability and agreement.

#### Intra-Rater Reliability

For each rater and each subject the rate of features that were evaluated identically was calculated.

#### Inter-Rater Agreement

For each subject and feature, the evaluations that appeared most often (mode) in the first evaluation of VRI images by the raters, were counted and specified as the number of agreements (frequency of mode = f (mode)). For all the features of each subject, the sum of f(mode) was calculated (Σf (mode)). Normalization to 0-100% agreement level was performed (0% - no agreement at all; 100% - full agreement), and the average inter-rater agreement was calculated (Equation (1)) [14-16] for all participants, both for healthy subjects and for subjects with pneumonia.(1)

i = 1...12 VRI features evaluated

j = 1....n subjects (overall n = 43, controls n = 20, pneumonia cases = 23)

The inter-rater agreement was analyzed using descriptive statistics, (one-way ANOVA; significance level 5%), and Intra-Class Correlation (ICC). ICC is a quadratically modified form of the Kappa correlation applied when multiple raters judge the same phenomena. A two-way random effects model (considering variables of VRI evaluated feature, raters) was used to calculate an averaged ICC by assuming that the ICC = ICC (subject, rater) for each VRI evaluated feature. For ICC results, positive values ranging from 0 to <0.2 indicate poor agreement, >0.2 to 0.4 fair agreement, >0.4 to 0.6 moderate agreement, >0.6 to 0.8 good agreement, and >0.8 to 1 very good agreement [14-16].

Normal distribution was assessed using the Shapiro-Wilk test. The independent samples T-test was applied for the comparison of approximately normally-distributed variables and the Mann-Whitney U test where there was evidence of non-normality and ANOVA was applied for multiple comparisons. Data analysis was carried out by using the SPSS statistical software package (SPSS Inc., Chicago, IL, USA; version 15.0). A p value < 0.05 was considered statistically significant.

## Results

### VRI findings

**Figure 2:**
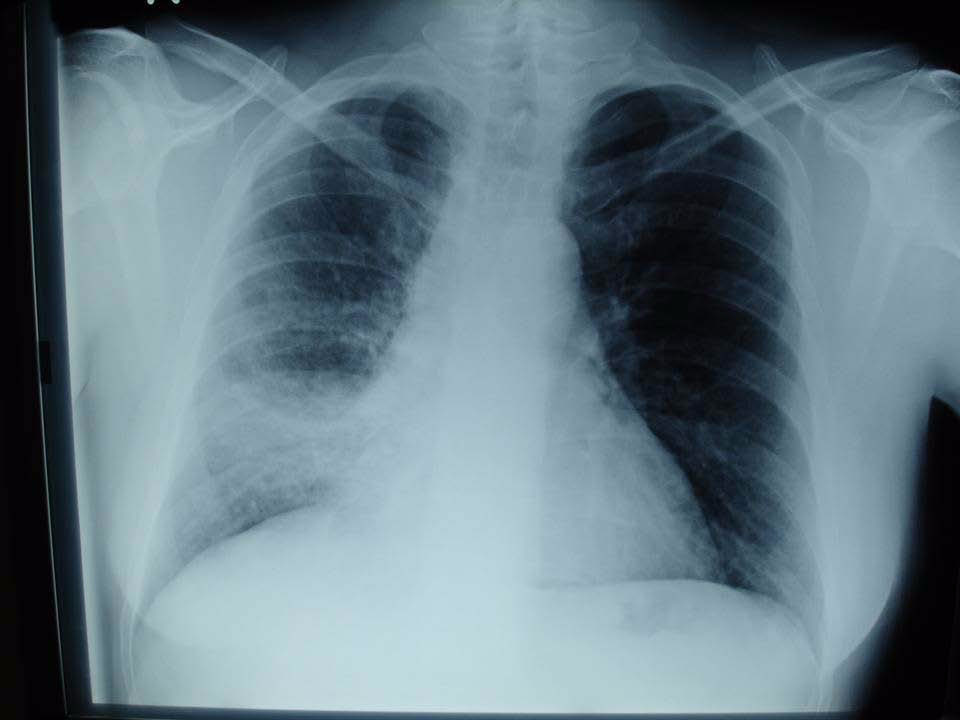
**Chest radiography from a patient with right lower lobe pneumonia**.

**Figure 3:**
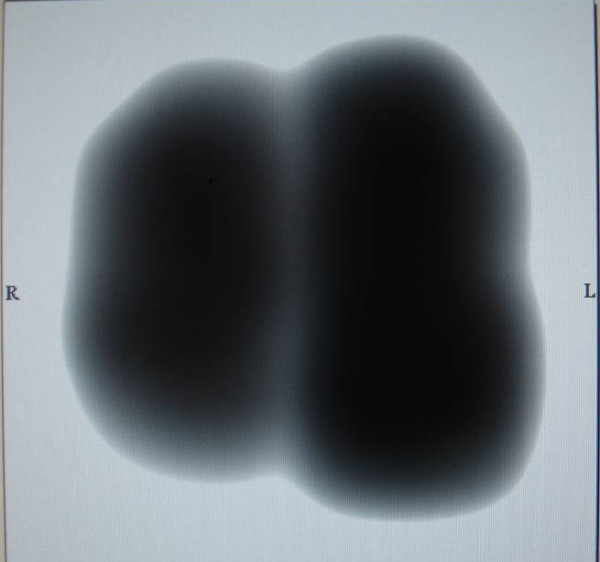
**Vibration response imaging (VRI) obtained from the same patient (Figure 2)**. VRI shows the breath sound intensity distribution at the maximum energy frame (MEF). The missing part in the right lower field shows the area of pneumonia.

Basic characteristics of patients who participated in the study are shown in Table 1. Among the patients with pneumonia 20 had Chronic Obstructive Pulmonary Disease (COPD), 8 arterial hypertension, 11 stable cardiac disease (congestive heart failure 5 cases, ischemic heart disease 6 cases) and 2 diabetes mellitus. There were eighteen cases with radiographically right lung consolidation (13 in the lower and 5 in the upper lung field) and 5 cases with left lung consolidation (3 in the upper lung field and 2 in the lower lung field). A representative case is shown in Figure 2 and 3.

**Table 1:** Characteristics of study participants

	Patients	Controls
Age (y)	69 (52-72)	52 (32-69)
Sex (F/M), n	3/20	7/13
BMI (kg/m2)	26.8 (25.9-28.7)	25.8 (25-26.5)
Carlson index	1 (1-3)	n/a
Pack-years	60 (40-90)	n/a
FVC, % predicted	79.5 (69-87.2)	n/a
FEV1, % predicted	67 (42.2-72.5)	n/a
FEV1/FVC, % predicted	67 (54-70)	n/a
pO2, mmHg	72 (61.5-80)	n/a
Pco2, mmHg	34.2 (29.5-38)	n/a

Data are presented as median (IQR) values otherwise is indicated

The mean (SE) time for completing a VRI recording in patients and controls was 8(2) and 6(2) minutes respectively (T-test, p = 0.2). Raters identified small artefacts (0.36 artefacts per image by each rater) in VRI images in both healthy (0.27 artefacts/image/rater) and pneumonia cases (0.41 artefacts/image/rater).

### Qualitative Interpretation of VRI images

#### Intra-Rater Reliability

The average value for overall identical evaluations of twelve features of the VRI image evaluation by the raters, ranged from 87% to 95% per rater (94% to 97% per rater in control cases and from 79% to 93% per rater in pneumonia cases). The percentage of identical evaluations for each individual feature evaluated by raters demonstrated a high rate of consistency ranging from 87% to 95% (92% to 98% in control cases and 83%-92% in pneumonia cases). Overall, the feature interpreted with the highest intra-rater agreement was an abnormally decreased signal in the right lower zone (95%) whereas a decreased signal in the left lower zone was interpreted with the lowest agreement (87%).

#### Inter-Rater Agreement

**Figure 4:**
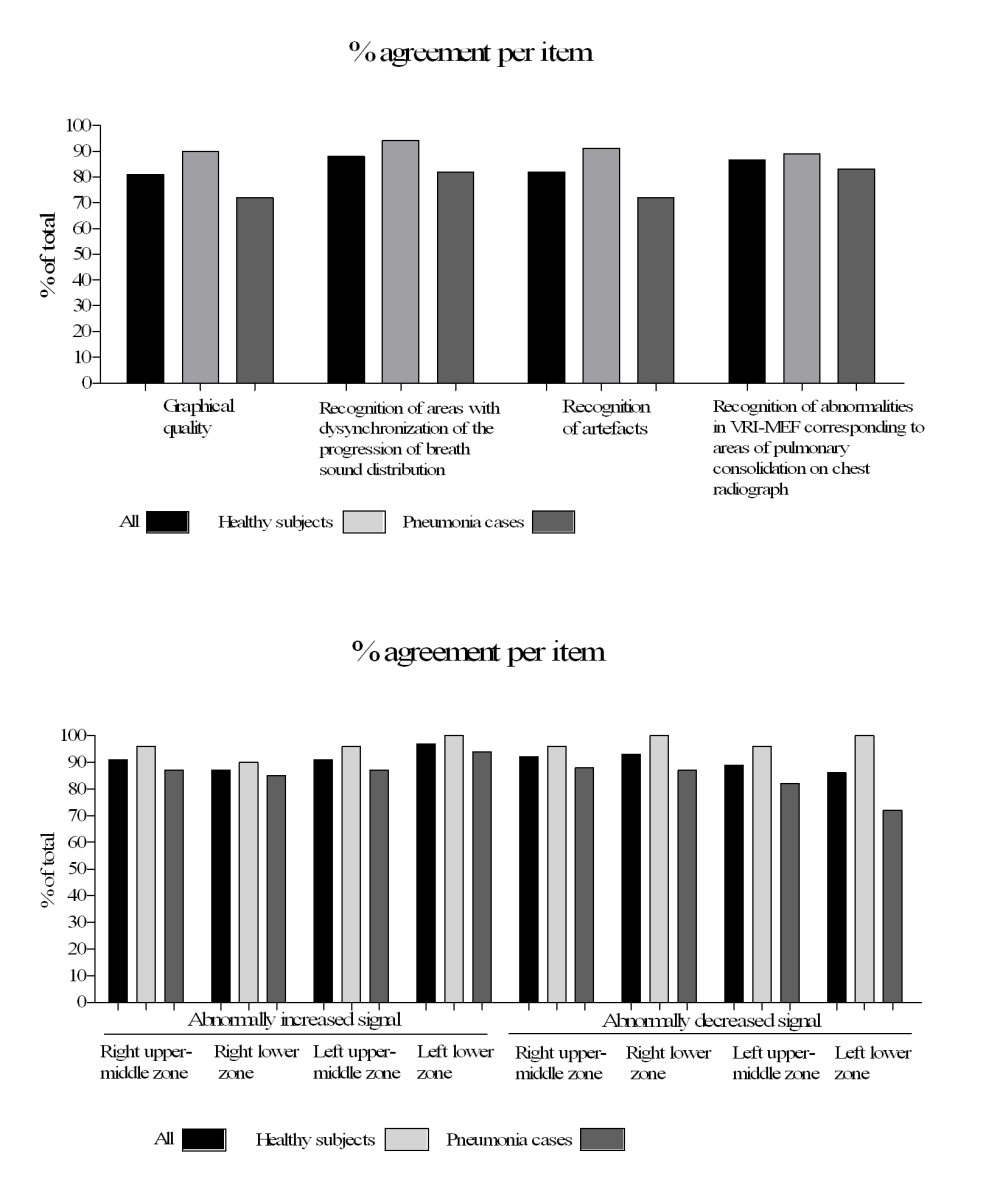
**Inter-rater agreement (%) per VRI feature evaluated**.

The median (IQR) agreement based on the images from all 43 participants (both normal and pneumonia cases) was 91% (82%-96%). The agreement was very good in both healthy subjects and pneumonia cases [94% (88%-100%) and 87% (73%-93%) respectively]. In addition, no significant difference was found in terms of inter-rater agreement when we split our sample into cases where either >3 or, ≤3 raters identified artefacts in VRI (T-test, p = 0.8). The level of agreement according to VRI feature evaluated was in most cases over 80%. Lower values were noted in features related to localization of abnormalities in pneumonia cases, but agreement was over 70% in all cases (Figure 4). ICC obtained by using a model of subject/rater for the averaged features, was overall 0.86 (0.92 in normal subjects and 0.73 in pneumonia cases).

## Discussion

The present prospective study evaluated intra- and inter-observer variation of VRI interpretation in a population consisted of healthy subjects and patients with pneumonia. The average value for overall identical evaluations of several VRI features evaluated by the raters, ranged from 87% to 95% per rater. In addition, our findings demonstrated very good agreement between different raters in the interpretation of VRI findings for both healthy subjects and patients; ICC for inter-rater agreement was 0.86. These results are in agreement with a previous study which assessed VRI repeatability in healthy subjects [7] and demonstrated very good repeatability of the method. In addition, our results support findings from other studies which evaluated the diagnostic value of VRI in a population with pneumonia and pleural effusion but data regarding the repeatability of the method were not reported. In this respect our investigation provides evidence suggesting that this novel method of imaging, which can be applied at the bedside, may be helpful in the management of patients with lung consolidation/atelectasis and deserves consideration.

In this study, we evaluated the technique in a population consisted of healthy subjects and patients hospitalized due to pneumonia. The average value for identical evaluations of VRI features evaluated by the raters, ranged from 94-97% per rater, in healthy control cases. The average inter-rater agreement, based on the images from normal cases was 91% and ICC was 0.86. This is in accordance with a previous investigation [7] that assessed extensively the intra- and inter-rater agreement of reviewers in the interpretation of VRI in healthy subjects and reported good levels of agreement and consistency. Maher et al [7] assessed the reproducibility of VRI using recordings from 29 healthy individuals, on three separate time points, evaluating several VRI features - some of them were similar to features evaluated in the present study. In that study [7] the average value for identical evaluations of VRI features evaluated by the raters, ranged from 88% to 95% per rater and ICC for inter-rater agreement was 0.61. Despite differences in methodology between the present and Maher's study [7], the results of qualitative assessment of VRI in healthy individuals in both studies are comparable and suggest good to very good inter-rater agreement and consistency in VRI images interpretation.

In the present study we studied the agreement between physicians in both healthy subjects and patients with pneumonia and the intra- and inter-rater agreement was good. VRI is a novel method and data regarding the interpretation of VRI images and the reproducibility of the method are sparse. Earlier computational adventitious lung sound analysis studies showed the potential of this method for diagnosing lung pathology [4,17]. The utility of the technique has been demonstrated in clinical cases where dynamic interventions are taken place such as in interventional pulmonology and critical care [8,9,12]. In addition, another study has shown that changes in ventilation have a discernible and reproducible effect on the pattern and distribution of dynamic acoustic lung images in the intensive care setting [9]. However, another study [18] has demonstrated poor inter-observer agreement in the detection of abnormal respiratory noises in infants. In addition, while Mor et al [12] studied a mixed population of patients with pneumonia and pleural effusions, the reproducibility of the method was not noted. Thus, definitive conclusions for the reproducibility of the technique, especially in the setting of specific disorders have not been reached.

In our study, we found that agreement on the presence of abnormally high or low intensity on VRI-MEF corresponding to the areas of pulmonary consolidation on chest radiographs or absence of an abnormality on chest radiographs was >80%. Furthermore, we found good agreement on localisation of abnormalities in pneumonia cases (Figure 4). Thus, the present study provides evidence for the reproducibility of the method in the clinical setting and suggests that VRI could be helpful in the diagnosis and follow up of pneumonia.

In this investigation, we evaluated several aspects of VRI technology in order to provide useful data regarding this novel method. First, we noted that the presence of consolidation/atelectasis which is expected in pneumonia might be related with abnormally increased or decreased signal in VRI although in most cases at the radiographic site of pneumonia there was a decreased intensity of the signal in VRI image. An abnormally decreased signal might indicate decreased ventilation in the lung region and thus, decreased breath sounds which are occasionally found together with other focal lung findings in areas of consolidation [19]. In fewer cases an increased intensity was identified in VRI, a sign which might have been produced due to vibrations produced by secretions. The overall agreement between raters regarding the detection of abnormal signals and the localization of abnormalities was good or very good. Thus, our study demonstrated that physicians may be able not only to detect the presence of an abnormality in VRI, but they might also be able to locate the abnormality.

It should be however noted that the agreement regarding decreased signals in specific lung zones, such as left lung zones, was lower than the average values. The most plausible explanation for this fact might be the interference of the heart in left lung fields which might make the interpretation of VRI more difficult. It should be also noted that quantitative analysis of VRI signal distribution, via the signal energy obtained by the regional sensor, has not been performed in this study. As a result, quantitative assessment of left to right distribution has not been performed and this is a limitation of the present study. Thus, we cannot exclude that the results for the left lower lobe might have been different from those for the right lung due to the different number of lobes in each lung.

Furthermore, it should be underlined that raters showed very good agreement for the presence of small artefacts in some cases in both healthy subjects and patients. One explanation for the presence of artefacts could be that they were artefacts created by unintentional direct outer contact of the operator to the sensor or by environmental noise. VRI is a real time imaging system which is based on sound analysis and artefacts might be one of its drawbacks in the everyday clinical setting, for the time being. We believe that artefacts could be obviated in the future with advances in sensor technology and software. On the other hand, we certainly cannot exclude that artefacts might also represent affected regions that could not be detected in simple radiography. Unfortunately, Computed Tomography which has higher sensitivity than chest radiography was not available in this study and therefore, we cannot rule out the possibility of the above. However, when we split our sample into cases where either >3 or ≤3 raters identified artefacts in VRI, inter-rater agreement between physicians remained similar. Thus, we believe that the presence of artefacts has not affected our results.

One might argue that we evaluated VRI images interpretation by physicians and radiologists were not included. In this respect, this could also question the reliability of the reference chest radiography evaluation for the final diagnosis of pneumonia. This might represent a limitation of our study, which we certainly acknowledge. Furthermore, we included a population of patients with comorbidities such as COPD and cardiac disease. The coexistence of other diseases, especially COPD, may affect lung sound distribution by adding artefacts or by altering the sound distribution and therefore might make the interpretation of VRI difficult. However, we intended to evaluate this novel method in the everyday clinical setting where patients with pneumonia have often co-morbidities, and thus the application of the method should also take this into account.

In the present study we compared directly the VRI-MEF image with chest radiography. We certainly acknowledge that VRI and radiography are two methods of imaging based on different principles. The former is dynamic and the latter is not. In this respect, one might argue that comparison between these methods is not appropriate. However, both are diagnostic imaging techniques which are based on the imaging properties of tissues containing air representing the status of the lungs at maximum inspiration. VRI is a technique of real time imaging which may improve clinical diagnosis since it provides data which might supplement information provided by simple auscultation [20]. In addition, VRI uses a multisensor device that simultaneously records lung sounds from 40 points over 12 seconds and the physician can be less dependent on memory. Furthermore, the technique does not involve radiation and thus, has an advantage over chest radiography especially in the follow up of pneumonia [11,21,22].

In this study, we assessed aspects of VRI technology in both healthy subjects and patients with pneumonia. It is true that pneumonia may coexist with atelectasis of pulmonary parenchyma or can be due to aspiration or a similar image can be the result of a local haemorrhage. Thus, VRI assessment in these different categories of lung parenchymal disorders could give further useful information for the clinical application of this method. Diagnostic studies such as Computed Tomography that could help further in distinguishing between pneumonia and coexisting disorders, (i.e. adjacent atelectasis) have not been performed systematically in our study. Thus, future studies could assess whether VRI could be diagnostically useful in distinguishing different types of pulmonary parenchymal disorders, by using CT data as a reference.

## Conclusion

Our findings suggest that the intra- and inter-rater agreement in the interpretation of VRI findings in both healthy subjects and patients with pneumonia is very good. Therefore, this device which is safe, radiation-free and user friendly may provide clinically important information to facilitate the diagnosis and follow up of common diseases such as pneumonia and has the potential to be applied as a complementary method to auscultation and chest radiography. Future investigations in larger cohorts and improvement in software and imaging analysis might refine further the method and may provide further data regarding the diagnostic value of VRI.

## Abbreviations

BMI: body mass index; CXR: Chest X-ray; COPD: Chronic Obstructive Pulmonary Disease; ICC: Intra-Class Correlation; FEF 25-75%: forced mid-expiratory flow; FEV1: forced expiratory volume in one second; FiO2: fraction of inspired oxygen; FVC: forced vital capacity; MEF: maximal energy frame; PaO2: partial arterial oxygen tension; PaCO2: partial arterial carbon dioxide tension; VRI: Vibration Response Imaging.

## Competing interests

The authors declare that they have no competing interests.

## Authors' contributions

KB drafted the manuscript, CD participated in data collection and study coordination, SP performed statistical analysis, PZ participated in data collection, AT participated in data collection, TK participated in data collection, DM participated in study design and reviewed the manuscript for important intellectual content, KIG and ZD participated in study design and motivated the study. All authors read and approved the manuscript.

## Pre-publication history

The pre-publication history for this paper can be accessed here:

http://www.biomedcentral.com/1471-2342/10/6/prepub
